# Category-based attention facilitates the recognition of actions

**DOI:** 10.1038/s41598-026-63368-4

**Published:** 2026-08-07

**Authors:** Federica Danaj, Lena Geßele, Jens Schwarzbach, Paul Downing, Angelika Lingnau

**Affiliations:** 1 Chair of Cognitive Neuroscience, University of Regensburg, Regensburg, Germany; 2 Department of Psychiatry and Psychotherapy, University of Regensburg, Regensburg, Germany; 3 Department of Psychology, Bangor University, Bangor, Wales

**Keywords:** Action recognition, Visual attention, Attentional templates, Category-based attention, Top-down attention, Psychology, Human behaviour

## Abstract

Action recognition relies on efficiently prioritizing relevant sources of information. This study examined the degree to which category-based attention facilitates the identification of basic action categories (climb, jump, run, walk) depicted as static avatar images (Exp. 1, 2) and naturalistic images (Exp. 3, 4). Participants received prior information about the target action category through a word cue (Exp. 1, 2, 4) or an avatar image cue (Exp. 3), with a 3:1 ratio of valid to invalid trials. Performance was assessed in terms of accuracy, reaction time (RT), and sensitivity (d-prime). Exp. 1 revealed a cueing effect of word cues on processing subsequent avatar stimuli that depicted different actions. Exp. 2 confirmed that this facilitation was due to the cue itself rather than specific motor associations. Exp. 3 indicated that avatar cues aid action recognition in naturalistic images, though the effect was weaker and limited to accuracy. Exp. 4 combined word cues with the same naturalistic images and revealed robust cueing effects on both accuracy and RT, indicating that top-down facilitation is preserved for naturalistic stimuli when word cues are used. Together, these findings provide an important extension of previous studies, suggesting that category-based attention enhances action recognition by pre-activating relevant action representations.

## Introduction

Accurately perceiving and interpreting human actions is vital for daily social interactions and communication. Our focus shifts based on our goals: for instance, learning a pirouette in ballet requires attention to movement execution, while an airport officer must quickly differentiate between walking and running to access security threats. Advance cues bias competition among different sources of information, enabling efficient event categorization and shaping predictions about upcoming sensory events^1–3^. Here, we examined how category-based attention enables us to focus on relevant aspects of an action while filtering out irrelevant information.

Action understanding is assumed to rely on the perception and integration of perceptual evidence coming from diverse sources of information, such as actors, body postures, movements, objects, scenes, and their relationships, and on a comparison of this perceptual evidence with stored representations of familiar actions^4^. These representations, in turn, can be manipulated with processes such as attention, enabling us to utilize the encoded information to inform and direct our behavior^5^. Investigating these interconnected levels offers a robust framework for uncovering the mental and neural mechanisms that underpin action recognition.

A useful approach to understanding how actions are represented draws on earlier research into the mental representation of objects^6–9^. The process of recognizing objects involves solving categorization challenges, which are similarly present in action recognition. For instance, both action and object recognition require generalizing across variations such as viewpoint, lighting, occlusion, and size while maintaining the specificity needed to differentiate between visually similar but distinct items^10^. Both domains also share a hierarchical organization within taxonomies, encompassing superordinate categories (e.g., “animals” or “locomotion”), basic-level categories (e.g., “birds” or “walking”), and subordinate categories (e.g., “robins” or “hiking”). These taxonomies enable flexible categorization, supporting the recognition of diverse exemplars while filtering out misleading candidates^11,12^.

Several authors propose that knowledge of objects is stored within a multidimensional representational space, where shared and distinguishing features define the proximity of items^8,9,13^. Similar principles have been suggested to apply to the representation of observed actions^12,14–19^. In this hypothetical multidimensional action space, each action occupies a specific point, and actions systematically vary along theoretical dimensions, reflecting properties like the involvement of a tool or whether the action targets another person.

By hypothesis, the distance between two actions in such a space directly correlates with their perceived similarity^15–17^. For example, running and walking appear close to each other, while walking and eating seem more dissimilar^15,16^. These action representations do not necessarily remain fixed; instead, cognitive processes are assumed to dynamically modulate them^4^. Selective attention has been suggested to play a critical role in this modulation, effectively shaping representational spaces to current needs by enhancing relevant dimensions at the expense of others^20–23^.

Attention can be directed to specific locations^24,25^, visual features^26^, or entire objects^27,28^ to prioritize sensory information, and to resolve competition between stimuli. This process has been suggested to be guided by attentional templates – preparatory representations that guide the selection and prioritization of task-relevant information by biasing neural competition^1^. While extensive research has aimed to distinguish the mechanisms underlying spatial, feature-based, and object-based attention, these differences often reflect the dominance of specific features in distinct cortical regions rather than fundamentally separate processes^29–31^. Recent studies propose representational structures as a unifying framework for attention, highlighting shared constraints and mechanisms that support efficient information processing across domains. This integrative perspective reconciles apparent distinctions among types of attention while emphasizing universal principles of selective attention^21,32,33^.

One of the most influential paradigms for guiding top-down attention involves the use of cues to activate the attentional template for the current target. These templates are assumed to encompass both high- and low-level visual features that best discriminate targets from non-targets^34^. Initially developed for spatial attention^24^, the classical cueing paradigm has since been adapted to explore non-spatial domains, demonstrating its versatility in studying various aspects of attentional control. This includes symbolic cues for the direction of upcoming motion of the target stimulus^35^ or symbolic letter-cues to search for objects categories in natural scenes (e.g., “people” or “cars”^36^). Likewise, word cues can target different features^37^ like orientation (e.g., “horizontal"^38^) or superordinate categories like “vehicle” or “animal"^39^. Research on object-based attention has found that valid cues enhance detection performance^39^. Moreover, prior work using word-cued visual search revealed that basic-level cues – such as *“*pants*”* within the broader superordinate category *“*clothing” – provide more precise and effective target templates than superordinate cues^40^, supporting the view that basic-level categorization plays a key role in object processing^11^.

Despite significant theoretical progress in the domain of object-based attention, the role of category-based attention in shaping the recognition of actions remains relatively underexplored. Many of the existing behavioral studies focused on “automatic imitation” tasks, which primarily investigate the links between action observation and execution^41–45^. While these studies provide valuable insights – suggesting that action understanding may not in fact be entirely automatic – they predominantly rely on highly simplified scenarios that may not fully capture the complexity of real-world action understanding. Specifically, they do not directly address the impact of attentional templates on behavioral performance in an action recognition task. By contrast, neuroimaging studies that examined the modulation of neural action representations via attentional templates lacked behavioral evidence^20,46,47^. To address the modulation of behavioral performance in action recognition via category-based attention, we designed a series of behavioral experiments targeting action recognition with a traditional attentional cueing paradigm from the object detection literature^39^.

Through these experiments, we aimed to test whether top-down preparation, enabled by an advance cue – either a word or an image indicating the upcoming action (climb, jump, run, or walk) – facilitates the identification of these basic action categories depicted as avatar images (Exp. 1, 2) and naturalistic images (Exp. 3, 4). We hypothesized that valid prior information about the action category of the target image through a word-cue (Exp. 1, 2, 4) or an avatar image (Exp. 3) would improve identification performance.

## Methods experiment 1

All procedures for this experiment were pre-registered on aspredicted.org (https://aspredicted.org/xbhd-ncpp.pdf). Experimental procedures were approved by the ethics committee at the University of Regensburg (Protocol 17–778_9-101), and all methods were performed in accordance with the relevant guidelines and regulations.

### Participants

Twenty-six healthy German native speakers with normal or corrected to normal vision participated in the experiment (*M*_age_ = 22.9, *SD* = 4.2, range = 19–38, 21 female). Participants were recruited among students at the University of Regensburg via SONA-Systems (https://www.sona-systems.com) and were compensated with credit points. All participants received written instructions about the experimental procedures and gave written consent to participate in the study.

### Apparatus

Participants were seated in front of a 21.5-inch screen (Dell P2219H, 1920 × 1080 resolution, 60 Hz) viewed from a distance of 80 cm while their head was stabilized by a chin rest. Stimulus presentation and data collection were implemented with A Simple Framework (ASF)^48^ based on the Psychtoolbox^49^.

### Stimulus material

We selected four basic level action categories (climb, jump, run, and walk) from the superordinate action category locomotion^12^, a key category in the cognitive structure underlying the organization of observed actions^15–17^ with ethological significance^50–52^. Basic-level actions were chosen as they represent the most salient level of action categorization^12^.

Avatar stimuli were created with Daz3D (Daz Productions, Salt Lake City, UT) and consisted of static images depicting a male or female 3D-Avatar with black uniform clothing performing actions on a gray background. To ensure participants relied solely on action information for the identification task, we intentionally excluded any background scene context, which could have introduced additional cues or distractions. Moreover, we chose static images over short animations to reduce motion-related complexities and focus participants’ attention more directly on the action categories. This design choice enabled a cleaner, more controlled assessment of category-based attention in action recognition, providing a robust foundation for future studies.

**Fig. 1:**
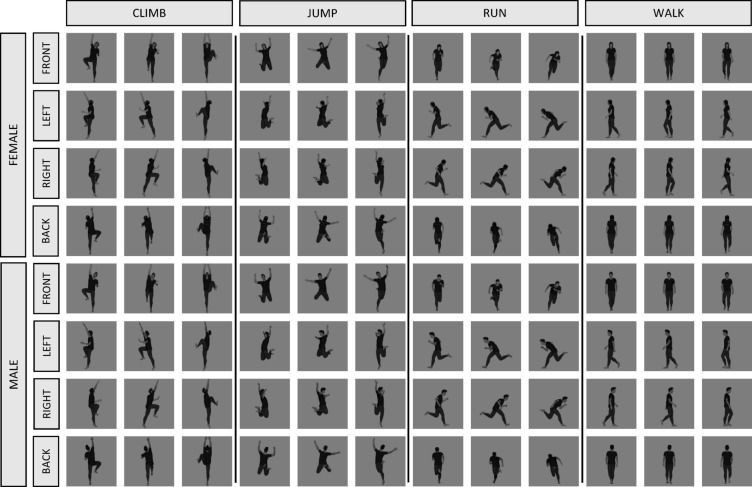
Illustration of the avatar stimulus set. The stimulus set consisted of four action categories (three exemplars each, shown in separate columns) depicting a female or male avatar from four different perspectives, for a total of 96 different stimuli.

Stimulus selection was based on an online survey (http://www.soscisurvey.com) with 100 action images. In this survey, *N* = 32 participants (*M*_age_ = 21.1, *SD* = 4.4, range = 18–39, 31 female) labeled and rated action images of female avatars viewed from four different perspectives (front, back, left, right) based on their typicality. In the first part, on each trial, participants were presented with an action image together with the question “Which action is represented in this image?“. They were asked to choose one of the following four options: climb, jump, run, or walk. In the second part, they rated the typicality of each image for the specified action category using a 7-point Likert scale (from 1 = very untypical to 7 = very typical). We selected 48 action images based on the following criteria: (1) they were mislabeled by less than 2 out of 32 participants, and (2) they had a typicality median value higher than 5. For the final stimulus set, we included the same 48 action images performed by male avatars, for a total of 96 stimuli, consisting of 4 action categories (climb, jump, run, walk) x 3 action exemplars x 4 perspectives (front, back, left, right) x 2 genders (female, male) (see Fig. 1).

Backward masks were inspired by Papeo et al.^53^. They consisted of high-contrast arrays (7.5°x7.5°) of randomly arranged grayscale circles of variable diameter (0.2°–1.9°). The grayscale values of these circles were chosen from the range of grayscale values of the avatars (0.068–0.5). The action images and the masks had a size of 400 × 400 pixels (visual angle: 6.9° x 6.9°).

Word cues (Verdana font, 35 points, white text color), action images, masks and a white fixation cross were centered on the screen and shown on a black background.

### Procedure

First, participants received written instructions about the experiment and provided written informed consent. Prior to the experiment, participants performed a training block consisting of 15 trials to familiarize themselves with the task.

**Fig. 2:**
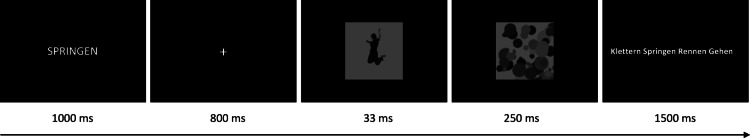
Schematic illustration of a valid trial for the target category “jump” (german: “springen”) in Experiments 1 and 2. The word cue (“climb”, “jump”, “run”, “walk”, or “ready”, german: “klettern”, “springen”, “rennen”, “gehen”, “bereit”) was followed by a fixation cross, the target image, a mask and the response screen.

Each trial began with a word cue (1000 ms) followed by a fixation cross (800 ms). Next, the target action image was briefly presented (33 ms) and immediately followed by a mask (250 ms; see Fig. 2 for an example trial). Afterwards, the response screen displaying the words “climb”, “jump”, “run”, and “walk” (German: “Klettern”, “Springen”, “Rennen”, and “Gehen”) was presented. The next trial started after 1500 ms. Participants were instructed to read the word cue, to focus on the fixation cross, and to select the word that described the previously shown action as quickly and accurately as possible by pressing the assigned keys “D,” “F,” “J,” or “K” on a keyboard placed in front of them, using their left and right index and middle fingers. The order of the keys on the keyboard matched the order of the words on the screen. The assignment of categories to keys was fixed for each participant and counterbalanced across participants. Participants were informed that the word cue correctly anticipated the action in most cases.

The experiment consisted of 768 trials: half were cued, and the other half were non-cued. In the non-cued trials, the word “ready” (“bereit” in German) was displayed. Among the 384 cued trials, the word cue validly predicted the target category in 2/3 of the trials (288 trials) by displaying the matching action category and invalidly predicted the target category in the remaining 1/3 (96 trials) by presenting one of the three non-matching action category words with equal probability. Throughout the experiment, each stimulus was presented 8 times. The order of trials was randomized, and 10 s breaks were introduced after every set of 96 trials. The total duration of the experiment was approximately 53 min.

### Data analysis

A two-factorial repeated-measures ANOVA was conducted, incorporating the within-subject factors *cue validity* and *action category* for each of the three dependent variables (accuracy, d-prime, and reaction time). *Cue validity* comprised 3 levels (valid, invalid, and non-cued), and *action category* comprised 4 levels (climb, jump, run, and walk), resulting in a 3 × 4 factorial design.

The first dependent variable, accuracy, was measured by the proportion of correct identifications (hit rate), while incorrectly selecting any of the other three action categories was labeled a “miss”. The second measure of accuracy, the discriminability index *d-prime (d’)*^54^, was calculated as the difference between the z-transformed hit rate and the z-transformed false alarm rate (d’ = z(hit rate) - z(false alarm rate)). The third dependent variable, reaction time (RT), was measured in milliseconds (ms) only for hit trials, measured from the onset of the response screen until a response key was pressed.

To gain a better understanding of the main effects and interactions, we computed post-hoc analyses using pairwise comparisons of the estimated marginal means (EMMs). Corrections for multiple comparisons were performed using Bonferroni correction separately for each of the three dependent measures and for all possible pairings of cue validity and action category. The reported p-values of the pairwise comparisons are Bonferroni-corrected. Effect sizes for the results of the ANOVA are reported as partial ^*2*^. When the assumption of sphericity was violated, as indicated by Mauchly’s test, we applied the Greenhouse-Geisser correction to adjust the degrees of freedom for the F-tests. Data analysis was performed using MATLAB (The MathWorks Inc., 2022) and SPSS (https://www.ibm.com/analytics/spss-statistics-software).

We pre-registered to exclude data from participants whose overall performance in any of the dependent measures was two standard deviations above or below the mean of the remaining participants.

## Results experiment 1

All participants fell within 2 standard deviations of the group mean and thus were included in the analysis. Accuracy and RT are reported below. Comprehensive ANOVA summaries for all dependent variables (d-prime, accuracy, and RT) across experiments are provided in the Supplemental Materials (Tables S1–S3). The corresponding d-prime results are shown in Fig. S1.

### Accuracy

**Fig. 3:**
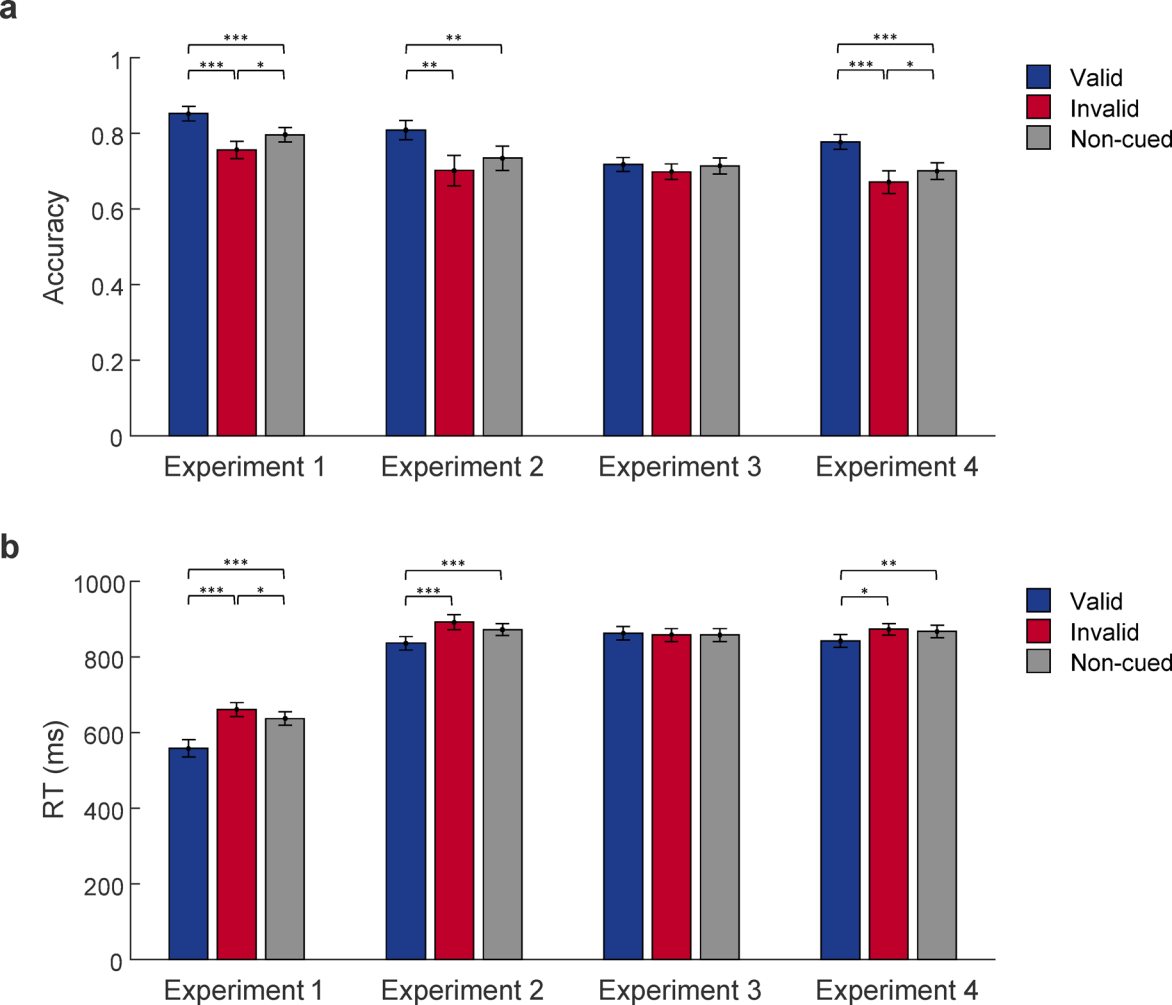
Mean accuracy **(a)** and RT **(b)** in Experiments 1–4 as a function of Cue Validity. Error bars indicate ± 1 SEM. Horizontal lines depict significant differences based on pairwise comparisons (*: *p* <.05; **: *p* ≤.01; ***: *p* ≤.001).

Figure 3A shows the mean accuracy as a function of cue validity. Accuracy was modulated by the validity of the cue (main effect of *cue validity: F*(1.22, 30.55) = 22.02, *p* <.001, *η*_*p*_^*2*^ = 0.47), with higher accuracy in valid (*M* = 0.85, *SEM* = 0.02) in comparison to invalid trials (*M* = 0.76, *SEM* = 0.02, *t*(25) = 5.05, *p* <.001) and in comparison to non-cued trials (*M* = 0.80, *SEM* = 0.02, *t*(25) = 6.91, *p* <.001). Likewise, accuracy was higher in non-cued in comparison to invalid trials (*t*(25) = 2.77, *p* =.03).

Accuracy was modulated by the category of the action (main effect for *action category*: *F*(1.83, 45.71) = 34.63, *p* <.001, *η*_*p*_^*2*^ = 0.58), with the highest mean accuracy for target images from the action category “walk” (*M* = 0.91, *SEM =* 0.02) followed by the action categories “run” (*M* = 0.86, *SEM* = 0.02), “climb” (*M* = 0.73, *SEM* = 0.03), and “jump” (*M* = 0.71, *SEM* = 0.03). Pairwise comparisons revealed that participants’ accuracy significantly differed between all action categories (*p* <.001) except for “climb” compared to “jump” (*t*(25) = 0.77, *p* = 1.0).

The effect of the cue on the accuracy was modulated by the action category (interaction *cue validity* X *action category*: *F*(6, 150) = 2.78, *p* =.014, *η*_*p*_^*2*^ = 0.1) (See Fig. S2). Pairwise comparisons revealed that accuracy was significantly higher for valid in comparison to invalid trials for all four action categories (climb: *t*(25) = 3.56, *p* =.005; jump: *t*(25) = 5.59, *p* <.001; run: *t*(25) = 3.77, *p* =.003; walk: *t*(25) = 3.27, *p* =.009), while non-cued trials did not differ from invalid trials in terms of accuracy in any of the four action categories (all *p* ≥.06). In comparison to the non-cued condition, accuracies in the valid condition were significantly higher for each action category (*p* ≤.001), except for “walk”, which did not show a significant difference (*p* =.32).

### Reaction time

Figure 3B shows the mean RT as a function of cue validity. RT was modulated by the validity of the cue (main effect of *cue validity*: *F*(1.36, 33.89) = 61.72, *p* <.001, *η*_*p*_^*2*^ = 0.71), with faster RT in valid (*M* = 558.64, *SEM* = 22.46) in comparison to invalid trials (*M* = 661.44, *SEM* = 18.48, *t*(25) = −8.22 *p* <.001) and in comparison to non-cued trials (*M* = 637.29, *SEM* = 17.98, *t*(25) = −11.15, *p* <.001). Likewise, RT was shorter in the non-cued trials in comparison to the invalid trials (*t*(25) = −2.79, *p* =.03).

RT was modulated by the category of the action (main effect of *action category* (*F*(2.31, 57.76) = 22.59, *p* <.001, *η*_*p*_^*2*^ = 0.47), with the fastest mean RT for target images from the action category “walk” (*M* = 551.94, *SEM =* 19.70) followed by the action categories “run” (*M* = 606.72, *SEM* = 20.90), “climb” (*M* = 653.42, *SEM* = 21.69), and “jump” (*M* = 664.42, *SEM* = 22.11). Pairwise comparisons revealed that participants’ RT significantly differed between all action categories (*p* ≤.004) except for “climb” compared to “jump” (*p* = 1.0) and “run” (*p* =.1).

Finally, the effect of the cue on the RT was not modulated by the action category (interaction *cue validity* X *action category*: *F*(4.36, 109.09) = 0.41, *p* =.82, *η*_*p*_^*2*^ = 0.02).

### Interim discussion

Experiment 1 showed that word cues enhance both the accuracy and speed of identifying basic action categories depicted as avatar images. Furthermore, invalid word cues were found to impair participants’ performance, with lower accuracy and RT in invalid trials compared to non-cued trials. These results suggest that the manipulation of top-down attention was effective, leading to improved performance. However, in Experiment 1, the order of the keys on the keyboard matched the order of the words on the screen, and the key assignment was fixed for each participant. This setup may have inadvertently cued specific motor responses rather than activating the semantic concept of the action category. To address this, in Experiment 2, we randomized the key assignments on each trial and for each participant.

## Methods experiment 2

Experiment 2 was designed to replicate the results of Experiment 1 while ensuring that no specific motor associations were cued. To achieve this, we randomized the assignment between response keys and action categories for each trial.

### Participants

Twenty-six healthy German native speakers with normal or corrected to normal vision participated in the experiment (*M*_age_ = 20.5, *SD* = 4.5, range = 18–41, 23 female). Participants were recruited among students at the University of Regensburg via SONA-Systems (https://www.sona-systems.com) and were compensated with credit points. All participants received written instructions about the experimental procedures and gave written consent to participate in the study. Experimental procedures were approved by the ethics committee at the University of Regensburg (Protocol 17–778_9-101).

### Apparatus

All the parameters of the apparatus were identical to those in Experiment 1.

### Stimulus material

Stimuli consisted of the same 96 avatar images used in Experiments 1.

### Procedure

The only difference between Experiment 1 and Experiment 2 was the assignment between response keys and action categories. In Experiment 2, this assignment was randomized for each trial, and the arrangement of the keys on the keyboard mirrored the order of the words on the screen. Participants were instructed to select the word describing the target action by pressing the key associated with its position.

### Analysis

We carried out the same analysis as described in Experiment 1.

## Results experiment 2

All participants fell within 2 standard deviations of the group mean and thus were included in the analysis.

### Accuracy

As can be seen in Fig. 3A, accuracy was modulated by the validity of the cue (main effect of *cue validity: F*(1.15, 28.75) = 9.96, *p* ≤.003, *η*_*p*_^*2*^ = 0.28). Pairwise comparisons revealed that participants’ accuracy was significantly higher in the valid (*M* = 0.80, *SEM* = 0.03) in comparison to the invalid condition (*M* = 0.70, *SEM* = 0.04, *t*(25) = 3.25, *p* =.01) and the non-cued condition (*M* = 0.73, *SEM* = 0.03, *t*(25) = 3.21, *p* =.01), but not significantly higher in the non-cued condition in comparison to the invalid condition (*t*(25) = 2.37, *p* =.08).

Accuracy was modulated by the category of the action (main effect of *action category*: *F*(2.18, 54.46) = 20.73, *p* <.001 *η*_*p*_^*2*^ = 0.45). Pairwise comparisons revealed that participants’ accuracy significantly differed for all factor levels (*p* ≤.004) except for “climb” compared to “jump” (*p* = 1.0), with the highest mean accuracy for target images from the action category “walk” (*M* = 0.86, *SEM =* 0.03), followed by “run” (*M* = 0.79, *SEM* = 0.03), “jump” (*M* = 0.68, *SEM* = 0.04), and “climb” (*M* = 0.67, *SEM* = 0.03).

The effect of the cue on the accuracy was modulated by the category of the action (interaction *cue validity* X *action category*: *F*(6, 150) = 4.05, *p* <.001, *η*_*p*_^*2*^ = 0.14) (See Fig. S3). Pairwise comparisons revealed that participants’ accuracy was significantly higher in valid compared to invalid trials for “climb” (*t*(25) = 3.57, *p* =.004) and “jump” (*t*(25) = 3.13, *p* =.01) action categories. However, there were no significant differences in accuracy for “run” (*t*(25) = 2.51, *p* =.06) and “walk” (*t*(25) = 1.9, *p* =.21) categories. Further, for all four action categories accuracy was not significantly higher for the non-cued compared to the invalid condition (*p* >.09). In comparison to the non-cued condition, accuracy in the valid condition was significantly higher for each action category (*p* <.035), except for “walk” (*t*(25) = 2.13, *p* =.13).

### Reaction time

As can be seen in Fig. 3B, RT was modulated by the validity of the cue (main effect of *cue validity*: *F*(2, 50) = 12.32, *p* <.001, *η*_*p*_^*2*^ = 0.33). Pairwise comparisons revealed that participants were significantly faster in valid (*M* = 843.45, *SEM* = 17.14) in comparison to invalid (*M* = 882.67, *SEM* = 16.99, *t*(25) = −4.33, *p* =.001) and non-cued trials (*M* = 880.46, *SEM* = 15.48, *t*(25) = −4.83, *p* <.001), but not significantly faster in non-cued compared to invalid trials (*t*(25) = −2.09, *p* = 1.0).

RT was modulated by the category of the action (main effect of *action category* (*F*(3, 75) = 16.33, *p* <.001, *η*_*p*_^*2*^ = 0.40). Pairwise comparisons revealed that RT was significantly different between action categories (*p* ≤.03) except for “climb” compared to “run” (*p* = 1.0) and “jump” compared to “walk” (*p* =.16), with the fastest mean RT for target images from the action category “walk" (*M* = 828.79, *SEM =* 19.32), followed by “jump” (*M* = 856.51, *SEM* = 16.42), “climb” (*M* = 888.24, *SEM* = 15.64), and “run” (*M* = 901.90, *SEM* = 17.35).

The effect of the cue on RT was modulated by the category of the action (interaction *cue validity* X *action category*: *F*(6, 150) = 0.41, *p* =.012, *η*_*p*_^*2*^ = 0.10) (See Fig. S4). Pairwise comparisons revealed that for most action categories participants’ RT was significantly faster in valid in comparison to invalid trials (climb: *t*(25) = −4.13, *p* =.001; run: *t*(25) = −4.38, *p* =.002; walk: *t*(25) = −3.07, *p* =.02), except for “jump” (*t*(25) = −1.55, *p* =.52). Further, for all four action categories RT was not significantly shorter for the non-cued trials in comparison to the invalid trials (*p* ≥.1). In comparison to the non-cued trials, RT in the valid trials was significantly shorter for each action category (*p* ≤.019), except for “walk” (*t*(25) = −2.54, *p* =.053).

### Interim discussion

To exclude the possibility of cueing specific motor associations, Experiment 2 introduced a randomization of the assignment between response keys and action categories on each trial. This control experiment confirmed the facilitatory effect of word cues on avatar targets, with participants responding faster and more accurately in valid trials compared to non-cued trials. However, we did not replicate the inhibitory effect obtained in Experiment 1: accuracy in invalid trials was not significantly lower than in non-cued trials, nor was RT slower.

Together, these findings emphasize the importance of category-based attention in efficient action identification. Knowing the semantic category of an upcoming action allows for the pre-activation of relevant visual action representations, prioritizing them over irrelevant actions. This, in turn, facilitates the visual processing of specific action instances when they align with the word cue.

Next, we aimed to explore whether the facilitatory effect observed with word cues is robust across different cue modalities and more naturalistic visual stimuli. Specifically, in Experiment 3, we examined whether image cues, instead of word cues, would produce similar benefits in action identification. An image cue, in principle, provides additional routes to support attentional selection of relevant action dimensions. To the extent that observers spontaneously apply a categorical label to the image cue, this may in turn function similarly to a word cue. At the same time, to the extent that there are image features in common between cue and target, even if they are not necessarily readily labeled or categorized, this overlap may also, where present, facilitate action categorization.

Finally, we sought to determine whether this effect would extend from avatar target images, used in Experiments 1 and 2, to more naturalistic target images depicting human agents performing actions.

## Methods experiment 3

In Experiment 3 we aimed to determine whether the facilitatory effect of top-down attention was generalizable to a different type of cue (avatar instead of word cue) and different target stimuli (naturalistic images instead of avatars).

### Participants

Twenty-six healthy German native speakers with normal or corrected to normal vision participated in the experiment (*M*_age_ = 22.6, *SD* = 3.4, range = 18–32, 21 female). Participants were recruited among students at the University of Regensburg via SONA-Systems (https://www.sona-systems.com) and were compensated with credit points. All participants received written instructions about the experimental procedures and gave written consent to participate in the study. Experimental procedures were approved by the ethics committee at the University of Regensburg (Protocol 17–778_9-101).

### Apparatus

All the parameters of the apparatus were identical to those in Experiment 1 and 2.

### Stimulus material

**Fig. 4:**
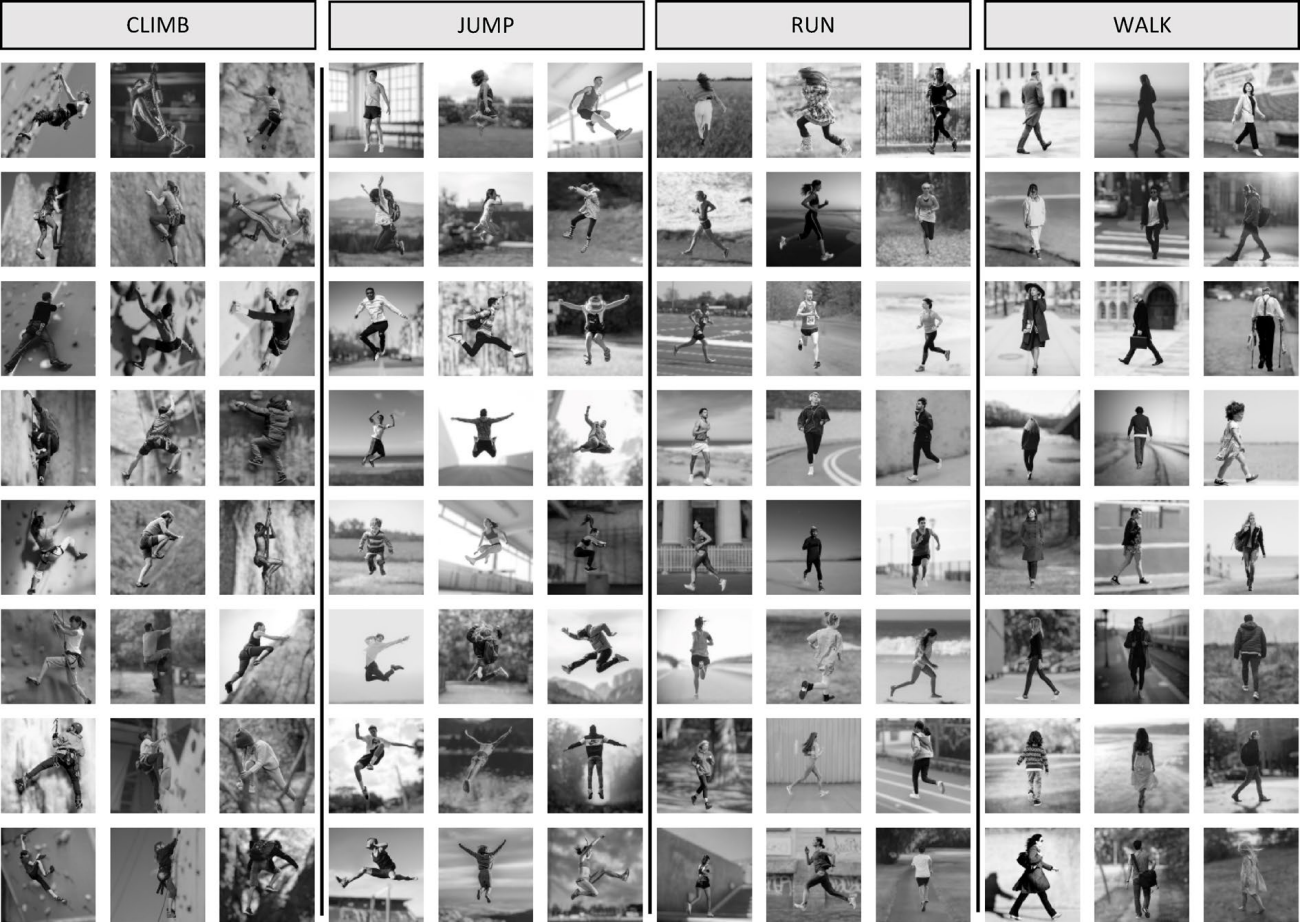
Illustration of the naturalistic stimulus set. The stimulus set consisted of static images depicting one of four action categories (climb, jump, run, walk), with 24 exemplars per category, for a total of 96 different stimuli.

Naturalistic stimuli consisted of grey-scaled pictures depicting people performing four basic actions belonging to the superordinate category locomotion. The stimuli were selected from different sources: Pexels (https://www.pexels.com), Shutterstock (https://www.shutterstock.com), Stanford 40 Action Dataset^55^, and Willow-Actions Dataset^56^. The final stimulus set (see Fig. 4) consisted of 4 action categories (climb, jump, run, walk), with 24 exemplars per action category (different age, gender, body position, and viewpoint).

The avatar cues consisted of the 96 static images of avatars performing actions used as stimuli in Experiment 1, along with two avatars (one male and one female) standing in a neutral position, viewed from 4 different perspectives (front, back, left, right), for a total of 104 avatar cues.

Backward masks consisted of scrambled images created by randomly selecting, shifting, rotating by 90 degrees, and shuffling 10 × 10 pixels squares from all naturalistic images. The avatar cues, action images and the masks had a size of 400 × 400 pixels (visual angle: 6.9° x 6.9°).

Avatar cues, action images, masks and a white fixation cross were centered on the screen and shown on a black background.

### Procedure

First, participants received written instructions about the experiment and provided written informed consent. Prior to the experiment, participants performed a training block consisting of 15 trials to familiarize themselves with the task.

**Fig. 5:**
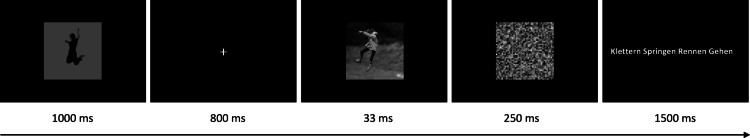
Schematic illustration of a valid trial for the target category “jump” in Experiment 3. The avatar cue (climb, jump, run, walk, or neutral) was followed by a fixation cross, the target image, a scrambled mask, and the response screen.

Figure 5 illustrates an example trial. Each trial began with an avatar cue (1000 ms) followed by a fixation cross (800 ms). Next, the target action image was briefly presented (33 ms) and immediately masked (250 ms). Afterwards, the response screen displaying the words “climb”, “jump”, “run”, and “walk” (German: “Klettern”, “Springen”, “Rennen”, and “Gehen”) was presented. The next trial started after 1500 ms. Participants were instructed to look at the avatar image, to focus on the fixation cross, and to select the word that described the previously shown action as quickly and accurately as possible. To do so, they had to press the assigned keys “D,” “F,” “J,” or “K” using a keyboard placed in front of them, where they positioned their index and middle fingers of both hands. The order of the keys on the keyboard matched the order of the words on the screen. The order of the words on the response screen were randomized for each trial. Participants were informed that the avatar cue correctly anticipated the action in most cases.

The experiment consisted of 768 trials: half were cued, and the other half were non-cued. In the non-cued trials, an avatar standing in a neutral position was displayed. Among the 384 cued trials, the avatar cue validly predicted the target category in 2/3 of the trials (288 trials) by displaying the matching action category, and invalidly predicted the target category in the remaining 1/3 (96 trials) by presenting one of the three non-matching action category images with equal probability (e.g., an avatar depicting running, followed by a target image depicting either climbing, jumping, or walking). Throughout the experiment, each stimulus was presented 8 times. The order of trials was randomized, and 10 s breaks were introduced after every set of 96 trials. The total duration of the experiment was approximately 53 min.

### Analysis

We carried out the same analysis as in Experiments 1 and 2.

## Results experiment 3

One participant performed below 2 standard deviations from the group mean RT; therefore, the dataset of this participant was excluded from the RT analysis.

### Accuracy

As can be seen in Fig. 3A, accuracy was modulated by the validity of the cue (main effect of *cue validity: F*(2, 50) = 3.69, *p* =.032, *η*_*p*_^*2*^ = 0.13). Pairwise comparisons revealed that participants’ accuracy was not significantly higher in the valid compared (*M* = 0.72, *SEM* = 0.18) to the invalid condition (*M* = 0.70, *SEM* = 0.02, *t*(25) = 2.54, *p* =.05), and the non-cued condition (*M* = 0.71, *SEM* = 0.02, *t*(25) = 0.62, *p* = 1.0), and not significantly higher in the non-cued condition compared to the invalid condition (*t*(25) = 1.77, *p* =.27).

Accuracy was modulated by the category of the action (main effect of *action category*: *F*(3, 75) = 40.72, *p* <.001, *η*_*p*_^*2*^ = 0.62). Pairwise comparisons revealed that participants’ accuracy significantly differed for all action categories (*p* ≤.005) except for “climb” compared to “jump” (*p* =.58). Mean accuracy was the highest for trials with target images from the action category “walk” (*M* = 0.82, *SEM =* 0.02) followed by “run” (*M* = 0.73, *SEM* = 0.02), “climb” (*M* = 0.66, *SEM* = 0.03), and “jump” (*M* = 0.63, *SEM* = 0.02).

Finally, the effect of the cue on the accuracy was not modulated by the category of the action (interaction *cue validity* X *action category F*(6, 150) = 0.93, *p* =.47, *η*_*p*_^*2*^ = 0.04).

### Reaction time

As can be seen in Fig. 3B, RT was not modulated by the validity of the cue (main effect of *cue validity*: *F*(1.4, 32.9) = 0.70, *p* =.45, *η*_*p*_^*2*^ = 0.28).

RT was only modulated by the category of the action (main effect of *action category*: *F*(3, 72) = 22.29, *p* <.001, *η*_*p*_^*2*^ = 0.48). Pairwise comparisons revealed that RT was significantly different for all action categories (*p* <.03) except for “climb” compared to “walk” (*p* = 1.0). Participants responded faster in trials with target images from the action category “walk” (*M* = 826.42, *SEM =* 17.27), followed by “climb” (*M* = 837.21, *SEM* = 18.53), “jump” (*M* = 886.51, *SEM* = 15.67), and “run” (*M* = 929.12, *SEM* = 16.32).

Finally, the effect of the cue on the RT was not modulated by the category of the action (interaction *cue validity* X *action category: F*(3.7, 88.8) = 0.43, *p* =.77, *η*_*p*_^*2*^ = 0.02).

### Interim discussion

Experiment 3 aimed to investigate whether the facilitatory effect of word cues observed in Experiments 1 and 2 could be generalized to a different type of cue (avatar cue) and a different type of target (naturalistic images).

While participants’ accuracy was significantly influenced by the validity of the cue, this effect was small and did not survive correction for pairwise comparisons. One possible reason underlying this observation might be the complexity of the target image, which might have required longer presentation times to be processed. In line with this view, accuracies for non-cued trials in Experiment 3 (M = 0.71, SEM = 0.02) were slightly lower in comparison to Experiment 2 (M = 0.73, SEM = 0.03). We thus cannot rule out the short presentation time chosen for the target images was not ideal to maximally benefit from the avatar cue.

As Experiment 3 did not allow us to determine whether the reduced facilitation was due to the cue type (avatar cue) or the complexity of the target (naturalistic images), we designed Experiment 4 to disentangle these potential sources by combining a word cue with the same naturalistic image set.

## Methods experiment 4

In Experiment 4, we aimed to disentangle whether the reduced facilitation observed in Experiment 3 was due to the cue type or the complexity of the target stimuli. To do so, we used word cues (as in Experiments 1 and 2) while keeping the target stimuli identical to Experiment 3 (naturalistic images).

### Participants

Twenty-six healthy German native speakers with normal or corrected to normal vision participated in the experiment (*M*_age_ = 20.5, *SD* = 2.1, range = 18–26, 19 female). Participants were recruited among students at the University of Regensburg and were compensated with credit points. All participants received written instructions about the experimental procedures and gave written consent to participate in the study. Experimental procedures were approved by the ethics committee at the University of Regensburg (Protocol 17–778_9-101).

### Apparatus

All parameters of the apparatus were identical to those in Experiment 1, 2, and 3.

### Stimulus material

Stimuli consisted of the same 96 naturalistic images used in Experiment 3 (see Fig. 4). Word cues (Verdana font, 35 points, white text color) were used instead of avatar cues.

### Procedure

**Fig. 6:**
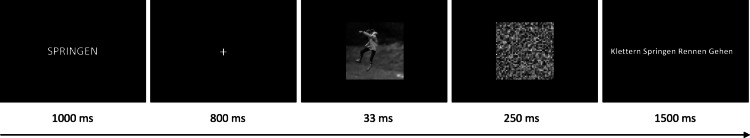
Schematic illustration of a valid trial for the target category “jump” (german: “springen”) in Experiment 4. The word-cue (“climb”, “jump”, “run”, “walk”, or “ready”, german: “klettern”, “springen”, “rennen”, “gehen”, “bereit”) was followed by a fixation cross, the target image, a scrambled mask and the response screen. .

Figure 6 illustrates an example trial. The only difference between Experiment 3 and Experiment 4 was the use of word cues instead of avatar cues.

### Analysis

We carried out the same analysis as in Experiment 1, 2, and 3.

## Results experiment 4

All participants fell within 2 standard deviations of the group mean and thus were included in the analysis.

### Accuracy

As can be seen in Fig. 3A, accuracy was modulated by the validity of the cue (main effect of *cue validity: F*(1.10, 27.41) = 18.21, *p* <.001, *η*_*p*_^*2*^ = 0.42). Pairwise comparisons revealed that participants’ accuracy was significantly higher in the valid (*M* = 0.78, *SEM* = 0.02) in comparison to the invalid condition (*M* = 0.67, *SEM* = 0.03, *t*(25) = 4.24, *p* <.001) and the non-cued condition (*M* = 0.70, *SEM* = 0.02, *t*(25) = 4.81, *p* <.001). Accuracy was also significantly higher in the non-cued condition than in the invalid condition (*t*(25) = 2.73, *p* =.04).

Accuracy was modulated by the category of the action (main effect of *action category*: *F*(2.43, 60.63) = 23.32, *p* <.001 *η*_*p*_^*2*^ = 0.45). Pairwise comparisons revealed that participants’ accuracy significantly differed for all factor levels (*p* <.02) except for “climb” compared to “jump” (*p* = 1.0). Accuracy was highest for target images depicting the action category “walk” (M = 0.81, SEM = 0.02), followed by “run” (M = 0.74, SEM = 0.03), “climb” (M = 0.66, SEM = 0.03), and “jump” (M = 0.65, SEM = 0.02).

The effect of the cue on the accuracy was modulated by the category of the action (interaction *cue validity* X *action category*: *F*(3.7, 91.5) = 6.2, *p* <.001, *η*_*p*_^*2*^ = 0.20; see Fig. S5). Pairwise comparisons revealed that participants’ accuracy was significantly higher in valid compared to invalid trials for the action categories “climb”, *t*(25) = 4.55, *p* <.001, “jump”, *t*(25) = 2.61, *p* =.050, and “run”, *t*(25) = 3.50, *p* =.005. No significant difference between valid and invalid trials was observed for the category “walk”, *t*(25) = 2.36, *p* =.078. Furthermore, for all four action categories, accuracy did not differ significantly between the non-cued and invalid conditions (*ps* > 0.05). In comparison to the non-cued condition, accuracy in the valid condition was significantly higher for the action categories “climb”, “jump”, and “run” (*ts*(25) ≥ 4.60, *ps* < 0.001), but not for “walk” (*t*(25) = 0.21, *p* = 1.00).

### Reaction Time

As shown in Fig. 3B, RT was modulated by the validity of the cue (main effect of *cue validity*: *F*(1.37, 34.11) = 8.42, *p* =.003, *η*_*p*_^*2*^ = 0.25). Pairwise comparisons revealed that participants were significantly faster in valid (*M* = 842.66, *SEM* = 16.84) in comparison to invalid (*M* = 873.29, *SEM* = 15.37, *t*(25) = − 2.97, *p* =.02) and non-cued trials (*M* = 868.08, *SEM* = 16.26, *t*(25) = − 3.62, *p* =.004), but not significantly faster in non-cued compared to invalid trials (*t*(25) = − 0.87, *p* = 1.0).

RT was modulated by the category of the action (main effect of *action category* (*F*(3, 75) = 30.50, *p* <.001, *η*_*p*_^*2*^ = 0.55). Pairwise comparisons revealed that reaction times differed significantly between action categories (*ps* ≤ 0.044), except for the comparison between “walk” and “climb” (*p* = 1.0). Reaction times were fastest for target images depicting the action category “walk” (M = 820.97, SEM = 16.72), followed by “climb” (M = 828.76, SEM = 17.32), “jump” (M = 864.80, SEM = 14.42), and “run” (M = 930.86, SEM = 20.51).

Finally, the effect of the cue on the RT was not modulated by the category of the action (interaction *cue validity* X *action category: F*(6, 150) = 1.8, *p* =.094, *η*_*p*_^*2*^ = 0.07).

### Interim discussion

Experiment 4 was designed to determine whether the reduced cueing effects observed in Experiment 3 were driven by the type of the cue or by the complexity of the target stimuli. By using word cues combined with the same naturalistic images, this experiment allowed us to isolate the role of the cue while keeping the complexity of the targets constant. The results show clear facilitatory effects of cue validity on both accuracy and reaction times. Participants responded more accurately and faster in valid trials than in invalid and non-cued trials, replicating the pattern observed in Experiments 1 and 2. These findings suggest that the reduced facilitation observed in Experiment 3 is unlikely to be solely explained by target complexity. Instead, they point to the cue type as a critical factor for effective attentional guidance, with more robust cueing effects for word cues in comparison to avatar cues. We will return to this point in the General Discussion.

## General discussion

This study investigated how top-down attention aids in identifying basic-level actions. Across four experiments, we examined how category-based attention prioritizes the actions climb, jump, run, and walk, and how cue modality and target type affect this process. Our findings offer novel insights into the cognitive processes underpinning action understanding while also shedding light on the strengths and limitations of different cueing paradigms.

Experiments 1 and 2 demonstrated that word cues significantly enhance both accuracy and speed in identifying basic-level actions depicted as avatar images. This supports the notion that semantic pre-activation of action categories facilitates the visual processing of target actions. Our findings align with prior research on category-based attention, which posits that foreknowledge of a stimulus category primes its corresponding mental representations, enabling efficient prioritization of relevant incoming information^38–40^.

To rule out that the effects observed in Exp. 1 were due to cueing of the motor response associated with the cue, Exp. 2 employed randomized key assignments. Using this approach, we were able to replicate the main facilitatory effect of valid word cues on action recognition obtained in Exp. 1, while we obtained no evidence for an inhibitory effect. The latter might indicate that the inhibitory effect obtained in Exp. 1 was partly driven by motor cueing. That said, we cannot rule out that increased task difficulty due to the random key assignment in Exp. 2 diminished the strength or detectability of this effect.

In Exp. 3, we examined whether the facilitatory effects of word cues generalize to image cues and naturalistic target stimuli. While image cues showed some influence on participants’ accuracy, the effect was weaker, and reaction times were not significantly affected by cue validity. This raised the possibility that the reduced facilitation reflected increased perceptual demands imposed by the naturalistic images, rather than limitations of cueing per se.

Exp. 4 was designed to resolve this ambiguity by combining word cues with the same naturalistic target images used in Exp. 3. The results showed robust facilitatory effects of cue validity on both accuracy and reaction times, closely resembling the patterns observed in Exp. 1 and 2. These findings indicate that top-down attentional facilitation generalizes to complex, naturalistic stimuli, provided that the cue offers an explicit written description of the upcoming action category. Together, Experiments 3 and 4 suggest that the reduced facilitation observed with avatar cues cannot be fully attributed to target complexity, but instead highlights the importance of the format of the cue for effective attentional guidance.

It is possible that word and avatar cues differ in their temporal dynamics, with differences in the optimal cue-target interval at which they unfold their maximum facilitatory effect. Since we did not systematically vary the cue-target interval, the present design does not allow us to directly address this possibility. Therefore, care needs to be taken when interpreting the obtained differences between word and image cues. We hypothesize that word cues may activate abstract semantic representations or prototypical templates of an action category, facilitating preparatory processes and efficient target matching^38–40^. Conversely, image cues may primarily evoke visual representations^38^, like a body posture, which can facilitate recognizing actions with similar body postures. Moreover, it is possible that word cues activate semantic representations, which in turn evoke visual ones, while image cues may first trigger visual representations that then activate the corresponding semantic representations. Throughout the experiment, participants also likely improved in using the cues to identify key features that distinguish the actions within these specific stimulus sets. In sum, our experiments do not definitively identify which level of representation is influenced by the word and image cues. Further experiments are required to distinguish between these alternatives.

Note that while we found reliable cueing effects for run, climb, and jump across all four experiments, cueing effects were overall weaker and less robust for the action “walk”, as reflected in the interactions between cue validity and action category observed in Exp. 1–4. One possible explanation is that participants were generally faster and more accurate in identifying “walk” than the other three actions, likely due to the familiarity and frequency of occurrence of this action. This may have left less room for additional improvement through attentional templates. Crucially, this pattern does not alter the overall conclusion of our study, namely, that top-down attention facilitates the recognition of observed actions across different formats (avatars, naturalistic images), a range of different viewing angles, and exemplars. At the same time, our restriction to actions from the superordinate category locomotion represents a limitation. Future studies should incorporate a broader range of action categories to assess the extent to which the results obtained in the current study can be generalized to a wider range of actions.

Overall, the beneficial influence of category-based attention observed in our study resonates with findings from behavioral and neuroimaging studies in the object and scene domain. Behavioral research indicates that effective detection in natural scenes relies on attention, as both spatial and category-based attentional processes enhance detection performance^39^. Functional magnetic resonance imaging (fMRI), electroencephalography (EEG), and transcranial magnetic stimulation (TMS) studies also consistently demonstrate strong top-down modulation of neural activity in response to task-relevant stimuli within natural scenes^28,36,57,58^. In object-selective cortical regions, neural responses are enhanced for task-relevant objects, while they are weakened or suppressed for irrelevant ones^35,59^. This category-specific modulation occurs early in the perception timeline, shaping the initial categorical representation of scenes^60^. Furthermore, cue-related activity prior to stimulus onset has been shown to act as a category-diagnostic template, biasing subsequent perceptual processing in favor of the task-relevant category^36,61^.

Our behavioral findings align with these behavioral and neural observations on category-based attention. Importantly, it is now clear that this top-down influence is not limited to object or scene perception but extends to the action domain, where attentional mechanisms modulate representational structures to prioritize task-relevant categories.

Our study is also a starting point to address the gap between cognitive and neural approaches to action understanding. While behavioral experiments mainly focused on simple proxies like the automatic imitation task^42–45^, neural studies examined how task relevance dynamically shapes more complex action representations^20,62^. The current findings suggest that integrating these perspectives can provide a more comprehensive understanding of how attentional mechanisms operate across different levels of analysis.

Word cues, by facilitating action identification, might reshape representational geometries. This dynamic tuning of the representational space ensures that stimuli along task-relevant dimensions – such as specific locomotion categories – or close to this point in space are significantly weighted and emphasized, while differences along unattended dimensions or in other regions of space are expected to be minimized^4^. For example, when deployed to distinguishing between animal taxonomy and behaviour, attention selectively enhances distances between stimulus-evoked neural representations along dimensions that are relevant to behaviour^62^. Similarly, by hypothesis, attentional mechanisms that highlight specific locations in the representational space may also sharpen the peaks surrounding those points, decreasing the similarity between elements that do not match any key dimensions, according to the goals of the task at hand^22^.

Our findings highlight several avenues for future research. First, future research should explore the neural underpinnings of the facilitatory effects observed for word cues. For example, does neural activity evoked by word cues resemble patterns elicited by the actual action categories? And do these facilitatory effects translate into more distinct neural patterns for task-relevant categories? Integrating these behavioral findings with neural evidence might bridge cognitive theories of attention with the neural mechanisms supporting representational tuning.

Second, exploring the differential effectiveness of word and image cues in different behavioral paradigms could clarify why semantic cues were more effective in this study. For example, in a visual search task, participants might rely more heavily on bottom-up perceptual features when searching for a matching target. Here, image cues might be particularly effective by priming visual features – such as body posture or motion – that align with the target. Conversely, word cues, providing abstract semantic information, might be less effective in tasks requiring perceptually driven identification. Testing word and image cues in a visual search framework could reveal whether word cues work better in tasks demanding abstract categorization, while image cues might be better suited for matching specific, low-level perceptual features.

Finally, future work could address how attentional mechanisms interact with perceptual load. In high-load scenarios, such as busy visual environments, the efficacy of semantic cues might depend on their ability to compete with irrelevant stimuli. This line of research could refine our understanding of how attention operates in complex real-world, dynamic settings, such as surveillance or human-computer interaction, and this could provide practical insights into improving action recognition in applied contexts.

## Conclusion

This study highlights the critical role of top-down attention in action recognition, demonstrating how cues facilitate action identification by activating attentional templates. This behavioral evidence supports future neuroimaging studies to examine how these attentional processes are reflected in neural representations, offering deeper insights into the interplay between attention and action understanding in both cognitive and applied contexts.

## Data Availability

The datasets generated and analyzed in this study are available in OSF (https://osf.io/q96wg/).
